# Isn’t high school bad enough already? Rates of gender harassment and institutional betrayal in high school and their association with trauma-related symptoms

**DOI:** 10.1371/journal.pone.0237713

**Published:** 2020-08-19

**Authors:** Monika N. Lind, Alexis A. Adams-Clark, Jennifer J. Freyd

**Affiliations:** Department of Psychology, University of Oregon, Eugene, Oregon, United States of America; Universidad de Chile, CHILE

## Abstract

Germinal studies have described the prevalence of sex-based harassment in high schools and its associations with adverse outcomes in adolescents. Studies have focused on students, with little attention given to the actions of high schools themselves. Though journalists responded to the #MeToo movement by reporting on schools’ betrayal of students who report misconduct, this topic remains understudied by researchers. Gender harassment is characterized by sexist remarks, sexually crude or offensive behavior, gender policing, work-family policing, and infantilization. Institutional betrayal is characterized by the failure of an institution, such as a school, to protect individuals dependent on the institution. We investigated high school gender harassment and institutional betrayal reported retrospectively by 535 current undergraduates. Our primary aim was to investigate whether institutional betrayal moderates the relationship between high school gender harassment and current trauma symptoms. In our pre-registered hypotheses (https://osf.io/3ds8k), we predicted that (1) high school gender harassment would be associated with more current trauma symptoms and (2) institutional betrayal would moderate this relationship such that high levels of institutional betrayal would be associated with a stronger association between high school gender harassment and current trauma symptoms. Consistent with our first hypothesis, high school gender harassment significantly predicted college trauma-related symptoms. An equation that included participant gender, race, age, high school gender harassment, institutional betrayal, and the interaction of gender harassment and institutional betrayal also significantly predicted trauma-related symptoms. Contrary to our second hypothesis, the interaction term was non-significant. However, institutional betrayal predicted unique variance in current trauma symptoms above and beyond the other variables. These findings indicate that both high school gender harassment and high school institutional betrayal are independently associated with trauma symptoms, suggesting that intervention should target both phenomena.

## Introduction

Important work occurs in adolescence. In the midst of physical, neurological, and psychological changes, adolescents undertake the intertwined tasks of social learning and identity development. Adolescents must also develop future-oriented, goal-directed skills that allow them to assume adult roles. Adolescence is a sensitive period, second only to infancy in its plasticity and its potential for positive or negative inflection points [1]. In many ways high school is the “workplace” where the important work of adolescence occurs. This study endeavors to investigate whether gender harassment and institutional betrayal create a hostile work environment in high schools and interfere with healthy adolescent development.

Gender harassment, a subdomain of sex-based harassment, includes verbal and non-verbal expressions and actions that derogate a person or group based on their gender [2]. Importantly, gender harassment does not necessarily convey sexual interest to the harassed party; rather, gender harassment conveys derision and/or hostility [2]. Leskinen and Cortina captured gender harassment in five domains: sexist remarks, sexually crude or offensive behavior, gender policing, work-family policing, and infantilization [3].

Most of the research on gender harassment has focused on its prevalence among adults, especially in the workplace. In adult samples, experiencing gender harassment is associated with a host of negative outcomes, including decreased psychological and professional well-being [2]. Much less is known about gender harassment in adolescence. Landmark studies by the AAUW capture aspects of gender harassment in their broader measure of sex-based harassment, and the data suggest high prevalence of gender harassment for boys and girls [4]. Like most other studies of sex-based harassment in high school, however, the AAUW studies assign only a few survey items to gender harassment and they do not test gender harassment as its own construct in their models. Gender harassment in high school deserves more attention, with parallel aims of describing its prevalence and dimensions and investigating its relationship with negative outcomes.

Trauma-related symptoms comprise one such category of potential negative outcomes that merit investigation. Trauma-related symptoms include dissociation, somatic complaints, mood-related problems, sleep disturbance, and others [5]. Sex-based harassment is associated with trauma-related symptoms in adults and adolescents [6, 7]. Evidence suggests that in adults, frequent gender harassment is as strongly associated with trauma-related symptoms as occasional, more coercive sex-based harassment [8]. It stands to reason that frequent gender harassment in high school could have similar associations with increased trauma-related symptoms.

Another limitation of the existing literature on high school sex-based harassment is that it does not address the high school institution's response to the harassment. Though journalists contributed to the #MeToo movement by publishing widely on the ways in which schools betray students who report misconduct [9, 10], this topic remains understudied by researchers. Institutional betrayal is characterized by the failure of an institution, such as a school, to protect individuals dependent on the institution [11]. Germinal studies have documented the occurrence of institutional betrayal across diverse contexts, including health care, the military, and college campuses [12–14]. These studies suggest that institutional betrayal may exacerbate the deleterious effects of stressful events.

Although these studies have provided foundational results regarding the prevalence and potential consequences of institutional betrayal, they have conceptualized institutional betrayal as only occurring in the aftermath of a traumatic event. This manifests in the studies’ designs in that only participants who reported experiencing a traumatic event were asked to report on their experience of institutional betrayal. Evidence of negative outcomes related to witnessing gender harassment and the normalization of sex-based harassment by schools suggests an alternative conceptualization of institutional betrayal [15, 16]. As the foundational literature indicates, institutional betrayal can be incident-specific, but it also may occur at the climate level.

The prevalence and dimensions of gender harassment in high school deserve detailed investigation, and the experience of gender harassment may be connected with trauma-related symptoms. Furthermore, high schools as institutions may play an important role in the association between harassment and trauma-related symptoms. For these reasons, we conducted an empirical study investigating the rates and correlates of high school gender harassment and institutional betrayal reported retrospectively by current undergraduates.

Our primary aims were (1) to describe the rates of gender harassment and institutional betrayal in high schools and (2) to investigate whether institutional betrayal moderates the relationship between high school gender harassment and current trauma symptoms. In our pre-registered hypotheses, we predicted that high school gender harassment would positively predict current trauma symptoms and that institutional betrayal would moderate this relationship.

## Methods

### Participants

The Office of Research Compliance (Institutional Review Board) at the University of Oregon approved this study. Informed consent was obtained in writing. Participants were recruited over the course of two academic terms from an undergraduate Human Subjects Pool managed by a public university located in the northwest United States. The institution’s Human Subjects Pool consists of undergraduate students over the age of 18 from psychology and linguistics courses. Students receive course credit for completing studies, and they are permitted to end their study participation at any time. Because of the design of the Human Subjects Pool, participants are not aware of the topic of the research prior to signing up to participate, which protects against biased self-selection [17]. This process was approved by the institution’s Office of Research Compliance (Institutional Review Board), as evidence suggests that trauma research is considered minimal risk [18, 19].

The overall sample consisted of 535 participants. Of this sample, 67.8% (*n* = 363) identified as female, 31.4% (*n* = 168) identified as male, 0.6% (*n* = 3) identified as non-binary, and 0.2% (*n* = 1) preferred not to report gender. The majority of the sample (68.4%; *n* = 366) identified as Caucasian; 1.3% (*n* = 7) identified as American Indian/Alaskan Native, 15.7% (*n* = 84) identified as Asian, 2.3% (*n* = 12) identified as African American, 1.3% (*n* = 7) identified as Native Hawaiian/Pacific Islander, and 11.0% (*n* = 59) identified as Other. Participants’ ages ranged from 18 to 23, with an average age of 19.20 (*SD* = 1.36). Before data analysis, one participant was excluded due to extensive missing data (all survey items left blank). Another participant was removed due to a response pattern characterized by high leverage on both independent variables and outlier status on the dependent variable. Because of this response pattern combined with the participant’s nonsensical gender response, “11^th^ dimension camera” (a phrase which appears nowhere on the internet), we opted to censor this participant.

### Measures

#### Gender harassment

Gender harassment was measured using the 20-item Gender Experiences Questionnaire (GEQ) [3]. The GEQ assesses five dimensions of gender harassment: Sexist Remarks (4 items), Sexually Crude/Offensive Behavior (5 items), Infantilization (3 items), Work/Family Policing (4 items), and Gender Policing (4 items). Initially developed to measure women’s experiences of workplace gender harassment in the past year [3], the GEQ was modified for this study in two ways. Rather than reporting on experiences in the workplace, participants were instructed to report on experiences of gender harassing behaviors during high school perpetrated by anyone associated with their high school, including classmates, coaches, teachers, school staff, and administrators. Three items from the Gender Policing subscale were also adapted to be more appropriate for male and gender-nonconforming participants. For example, an item on the original scale asked participants how frequently they were “made to feel like less of a woman because you had traditionally masculine interests.” This item was altered to “made to feel like less of a man because you had traditionally feminine interests” for male participants and “made to feel like less of a person because your interests were gender-nonconforming” for gender-nonconforming participants.

Participants rated each item on a response scale ranging from 1 (“Never”) to 5 (“Very Often”). Items from each subscale were then summed and averaged to create composite subscale scores ranging from 1 to 5, where higher scores represent more exposure to the respective dimension of gender harassment. All 20 GEQ items were summed and averaged to create a global composite GEQ score ranging from 1 to 5, where higher scores represent more exposure to total gender harassment. In prior research, the GEQ global and subscale scores have demonstrated excellent reliability (α’s ranging from .78 to .93) and validity [3]. In this study, the overall scale demonstrated excellent reliability (*α* = .94). The reliability of GEQ items were roughly equivalent for both women and men. Among women, the overall GEQ scale (*α* = .95), Sexist Remarks subscale (*α* = .96), Sexually Crude/Offensive Behavior subscale (*α* = .91), Infantilization subscale (*α* = .92), Work/Family Policing subscale (*α* = .92), and Gender Policing subscale (*α* = .85) demonstrated excellent reliability. Among men, the overall GEQ scale (*α* = .92), Sexist Remarks subscale (*α* = .86), Sexually Crude/Offensive Behavior subscale (*α* = .85), Infantilization subscale (*α* = .89), Work/Family Policing subscale (*α* = .89), and Gender Policing subscale (*α* = .85) demonstrated similar reliability. For details on our confirmatory factor analysis of the GEQ subscales, please see S1 Table. The distribution of scores (*Skew* = 0.70, *Kurtosis* = 0.22) was within the acceptable ranges.

#### Institutional betrayal

Institutional betrayal was measured using the 12-item Institutional Betrayal Questionnaire (IBQ) [20]. The IBQ assesses participants’ experiences with a range of institutional actions or inactions (e.g., “creating an environment in which sexual harassment seem common or normal”; “not taking proactive steps to prevent sexual harassment”; “making it difficult to report sexual harassment”). The 12-item scale consists of seven items included in the original version of the IBQ, as well as five additional items used in later research [20, 21]. When beginning the questionnaire, participants were provided with the following instructions: “Please consider your high school. (If you attended multiple high schools, please consider the high school at which you spent the most time enrolled.) How much do the following statements apply to the high school's attitudes and policies around sexual harassment?”. Unlike prior research that used a dichotomous yes/no response format [20], IBQ items in this study were rated on a continuous response scale ranging from 0 to 3, where 0 corresponds to “Very False,” 1 corresponds to “Somewhat False,” 2 corresponds to “Somewhat True,” and 3 corresponds to “Very True.” All items were summed to create a total composite IBQ score ranging from 0 to 36, where higher scores represent more frequent institutional betrayal. In this study, the IBQ demonstrated excellent reliability (*α* = .94). The distribution of scores (*Skew* = 0.64, *Kurtosis* = -0.38) was within the acceptable ranges.

#### Trauma symptoms

Trauma symptoms were measured using the 40-item Trauma Symptom Checklist (TSC) [5]. The TSC assesses a diverse range of common posttraumatic symptoms, including headaches, memory problems, anxiety attacks, nightmares, sexual problems, and insomnia. Participants were instructed to rate how often they experienced each symptom in the past month, with response options ranging from 0 (“Never”) to 3 (“Often”). All 40 items were summed to create a total composite TSC score ranging from 0 to 120, where higher scores represent more frequent trauma symptoms. In prior research, the TSC has demonstrated excellent reliability (α = .90) and validity [5]. In this study, the TSC demonstrated excellent reliability (*α* = .93). The distribution of scores (*Skew* = 0.93, *Kurtosis* = 0.78) was within the acceptable ranges.

#### Demographic information

In a demographics questionnaire, participants were asked to report their age, gender, and race.

### Procedure

An online survey containing all study materials was created through Qualtrics. The link to the survey was distributed to participants in the Human Subjects Pool using SONA online software. After clicking a link that directed them to the Qualtrics survey, participants were required to undergo an informed consent process and indicate their consent to participate. Participants completed the survey on personal electronic devices, and they were provided with course credit as compensation. Upon completion of the survey, participants were presented with a debriefing form. All research procedures were approved by the institution’s Office of Research Compliance (Institutional Review Board).

### Data analysis

Data were analyzed using *R* Version 3.5.2 and *R* packages *stats* (Version 3.5.2), *tidyverse* (Version 1.2.1), *psych* (Version 1.8.12), and *e1071* (Version 1.7–0.1) [22–25]. In order to test our hypotheses, we used linear regression analyses in which gender harassment, institutional betrayal, and gender harassment x institutional betrayal predicted current trauma symptoms, with participant age, gender, and race included as covariates. Continuous independent variables were standardized. The analysis plan was pre-registered on the Open Science Framework prior to any data observation (available at: https://osf.io/3ds8k).

#### Missing data

At the item level, 63 out of 38,520 items were missing (0.16%). One participant out of 535 was missing age (0.18%). At the questionnaire level, we conducted the Little MCAR test, and it was not significant. Therefore, we assume the data are missing completely at random. Missing data were imputed using single imputation through *R* package *Amelia* (Version 1.7.5) [26].

## Results

### Descriptive results

In the sample, 96.7% of women and 96.4% of men reported at least one instance of gender harassment in high school; only 12 women and 6 men reported no harassment experiences (i.e., scored the lowest possible score on the GEQ). Additionally, 87.3% of women and 76.7% of men reported at least one instance of institutional betrayal in high school; only 46 women and 39 men did not report institutional betrayal experiences (i.e., scored the lowest possible score on the IBQ). Participants had an average score of 2.25 (*SD* = 0.81) on the GEQ, 10.17 (*SD* = 8.67) on the IBQ, and 24.67 (*SD* = 17.07) on the TSC. Separate means and standard deviations for each gender category are reported in Table 1.

**Table 1:** Means and standard deviations by gender (N = 535).

Gender	*n*	Gender Harassment		Institutional Betrayal		Trauma Symptoms	
		*M*	*SD*	*M*	*SD*	*M*	*SD*
1. Women	363	2.32	0.84	11.46	9.01	26.58	17.07
2. Men	168	2.11	0.69	7.29	6.90	20.24	16.10
3. Non-Binary	3	2.97	1.15	18.67	15.70	47.33	15.28
4. Prefer Not to Say	1	1.20	NA	1.06	NA	10.00	NA

*M* and *SD* are used to represent mean and standard deviation, respectively.

**Fig 1:**
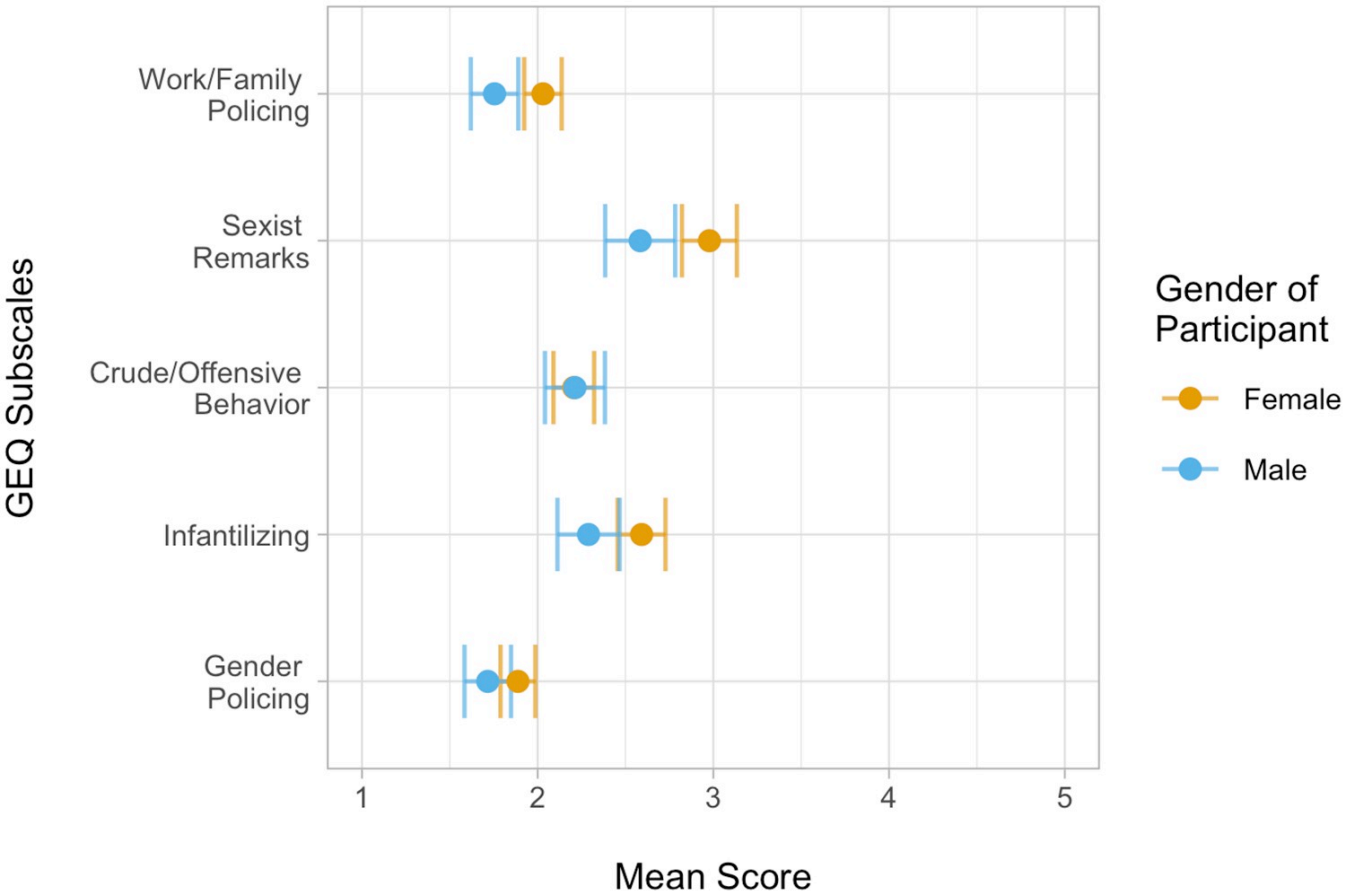
Mean GEQ subscale scores by gender of participant (N = 535; 363 women, 168 men) with standard error bars. Due to small cell size (*n* = 3), we excluded non-binary participants from this figure. Figure created using *R* packages *ggplot2* (Version 3.1.0) and *colorblindr* (Version 0.1.0) [27, 28].

Independent t-tests (with equal variances assumed) were conducted to assess gender differences in TSC scores, IBQ scores, GEQ scores, and the five GEQ subscale scores. In order to correct for the number of statistical tests, a Bonferroni correction was implemented, resulting in an adjusted alpha threshold of 0.0063. Because of low cell sizes, gender non-conforming (*n* = 3) participants were excluded from these analyses. Women had significantly higher scores than men on: the TSC, *t*(529) = 4.05, *p* < .001; the IBQ, *t*(529) = 5.32, *p* < .001; the overall GEQ, *t*(529) = 2.84, *p* = .005; the sexist remarks GEQ subscale, *t*(529) = 3.87, *p* < .001; the infantilization GEQ subscale, *t*(529) = 2.78, *p* = .006; and the work/family policing GEQ subscale, *t*(529) = 3.08, *p* = .002. Men and women’s scores did not significantly differ on the gender policing GEQ subscale, *t*(529) = 2.06, *p* = .04, or the sexually crude/offensive behavior GEQ subscale, *t*(529) = -0.07, *p* = .94. Men and women’s scores (and standard errors) on the GEQ subscales are displayed in Fig 1.

### Correlational results

**Fig 2:**
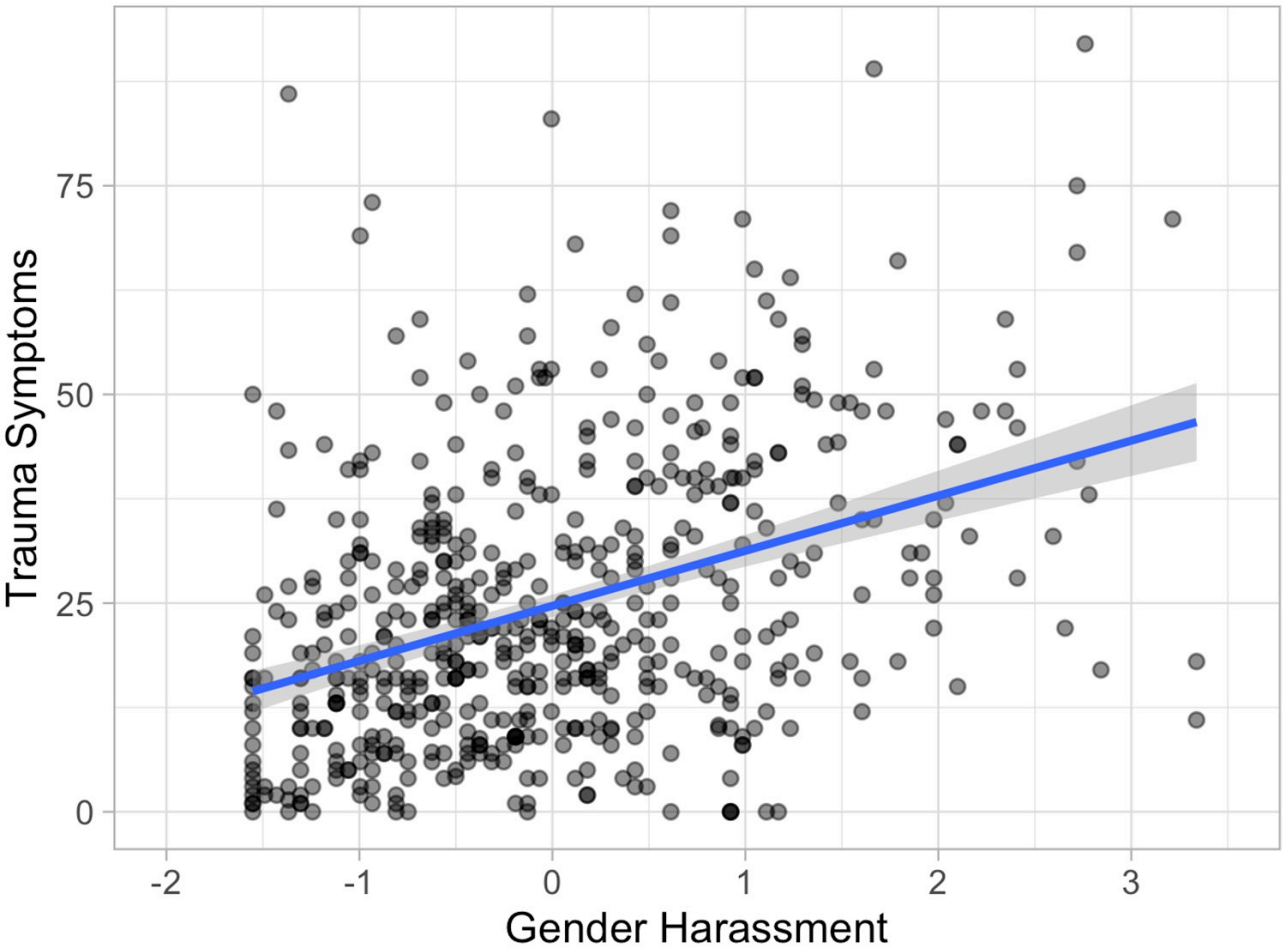
Relationship between trauma-related symptoms and high school gender harassment. Current trauma-related symptoms (raw TSC score) associated with self-reported high school gender harassment (standardized GEQ scores), plus line fit with “lm” method and 95% confidence interval. Figure created using *R* package *ggplot2* (Version 3.1.0) [28].

**Fig 3:**
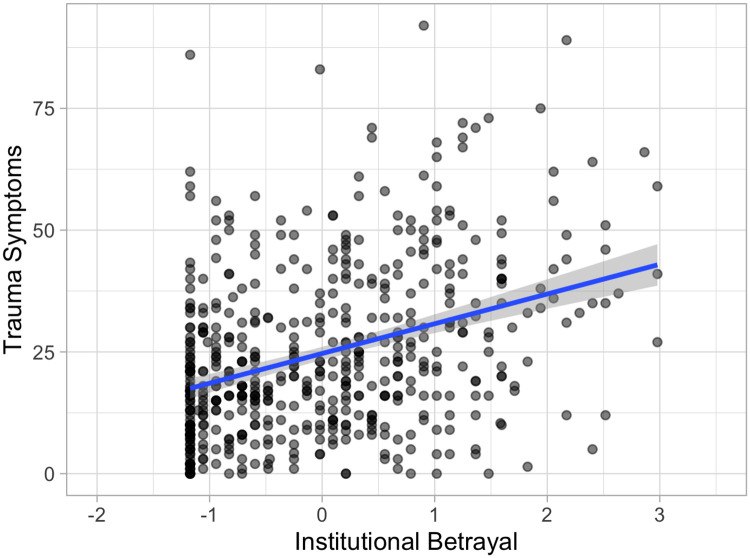
Relationship between trauma-related symptoms and high school institutional betrayal. Current trauma-related symptoms (raw TSC score) associated with self-reported high school institutional betrayal (standardized IBQ scores), plus line fit with “lm” method and 95% confidence interval. Figure created using *R* package *ggplot2* (Version 3.1.0) [28].

Pearson’s *r* bivariate correlation coefficients were calculated between continuous variables (see Table 2 for full correlation matrix). Significant positive associations were found between gender harassment, institutional betrayal, and trauma symptoms, *p* < .001. There was also a significant positive association between age and institutional betrayal, *p* < .01. The association between gender harassment scores on the GEQ and trauma symptom scores on the TSC are depicted in Fig 2. The association between institutional betrayal scores on the IBQ and trauma symptom scores on the TSC are depicted in Fig 3.

**Table 2:** Means, standard deviations, and correlations of continuous variables (N = 535).

Variable	*M*	*SD*	1	2	3
1. Age	19.30	1.36			
2. Gender Harassment	2.25	0.81	.03		
			[-.05, .11]		
3. Institutional Betrayal	10.17	8.67	.12**	.45***	
			[.03, .20]	[.38, .51]	
4. Trauma Symptoms	24.67	17.07	.01	.39***	.36***
			[-.07, .10]	[.31, .46]	[.28, .43]

### Multiple regression models

In order to test our main hypotheses, we used multiple regression to predict trauma symptoms (see Table 3 for complete regression table). In the first step of the model, we included participant age, gender (with female as reference group), and race (with Caucasian as reference group). These demographic characteristics together significantly predicted trauma symptoms, *F*(9, 525) = 3.61, *p* < .001, Δ*R*^*2*^ = .06. In this model, being male was associated with lower trauma symptoms, *b* = -6.69, *t*(525) = -4.24, *p* < .001, and identifying as “Other” race was associated with higher trauma symptoms, *b* = 5.00, *t*(525) = 2.11, *p* = .04. In the second step of the model, we included the main effects of gender harassment and institutional betrayal. A model comparison test indicated that the addition of these two predictors added significant predictive power to the model, *F*(2, 523) = 51.53, *p* < .001, Δ*R*^*2*^ = .16. In this step, both gender harassment, *b* = 4.63, *t*(523) = 6.15, *p* < .001, and institutional betrayal, *b* = 3.60, *t*(523) = 4.70, *p* < .001, were significant predictors of trauma symptoms. In the third step of the model, we included a gender harassment x institutional betrayal interaction term. A model comparison test indicated that the addition of this interaction term was not significant, *F*(1, 522) = 2.10, *p* = .15, Δ*R*^*2*^ = .003.

**Table 3:** Current trauma symptoms predicted by age, gender, race/ethnicity, gender harassment, institutional betrayal, and gender harassment x institutional betrayal (N = 535).

Predictor	*b*	*b*, 95% CI, [LL, UL]	Fit	Difference
Intercept	15.56	[-5.08, 36.21]		
Age	0.57	[-0.51, 1.65]		
American Indian/Alaskan Native	4.63	[-7.90, 17.16]		
Asian	-3.05	[-7.07, 0.96]		
African American	3.46	[-6.17, 13.10]		
Pacific Islander/Native Hawaiian	-1.89	[-14.43, 10.64]		
Other	5.00*	[0.35, 9.65]		
Male	-6.68***	[-9.78, -3.59]		
Non-Binary	18.36	[-0.78, 37.51]		
Prefer Not to Say	-23.63	[-57.01, 9.76]		
			*R*^*2*^ = .058**	
			95% CI[.01,.09]	
Intercept	27.04**	[7.93, 46.14]		
Age	-0.10	[-1.10, 0.90]		
American Indian/Alaskan Native	2.15	[-9.35, 13.65]		
Asian	-0.56	[-4.28, 3.16]		
African American	3.10	[-5.73, 11.92]		
Pacific Islander/Native Hawaiian	2.47	[-9.05, 13.98]		
Other	4.86*	[0.60, 9.12]		
Male	-3.48*	[-6.41, -0.55]		
Non-Binary	13.11	[-4.45, 30.67]		
Prefer Not to Say	-9.81	[-40.50, 20.88]		
Gender Harassment (GEQ)	4.63***	[3.15, 6.10]		
Institutional Betrayal (IBQ)	3.60***	[2.09, 5.10]		
			*R*^*2*^ = .213**	Δ*R*^*2*^ = .155**
			95% CI[.14,.26]	95% CI[.10, .21]
Intercept	26.96**	[7.87, 46.05]		
Age	-0.12	[-1.12, 0.88]		
American Indian/Alaskan Native	3.13	[-8.43, 14.70]		
Asian	-0.47	[-4.18, 3.25]		
African American	3.21	[-5.61, 12.03]		
Pacific Islander/Native Hawaiian	2.47	[-9.03, 13.98]		
Other	4.93*	[0.67, 9.19]		
Male	-3.50*	[-6.43, -0.58]		
Non-Binary	11.55	[-6.11, 29.22]		
Prefer Not to Say	-11.02	[-41.73, 19.68]		
Gender Harassment (GEQ)	4.49***	[3.00, 5.97]		
Institutional Betrayal (IBQ)	3.45***	[1.93, 4.96]		
GEQ x IBQ	0.97	[-0.35, 2.30]		
			*R*^*2*^ = .216**	Δ*R*^*2*^ = .003
			95% CI[.14,.26]	95% CI[-.01, .01]

## Discussion

Our results suggest that both gender harassment and institutional betrayal are common experiences in high school and that females report significantly higher rates of both than do males. Confirming our first pre-registered hypothesis, our results indicate that high school gender harassment is associated with college trauma-related symptoms. Contrary to our second pre-registered hypothesis, high school institutional betrayal did not moderate the relationship between gender harassment and trauma-related symptoms. Our findings did, however, show that institutional betrayal is associated with trauma-related symptoms above and beyond the predictive power of age, gender, race, and gender harassment.

Our study replicates three important findings. First, we found a high prevalence of gender harassment in high school, even higher than rates reported by the AAUW [4]. Second, we found a substantial relationship between the experience of sex-based harassment and negative outcomes, which is well established in the literature [29]. Third, we found that males reported experiencing less harassment than females, again mirroring a well-established pattern of results [30].

Our study bolsters the reliability and extends the utility of two recently developed measures. We successfully adapted and deployed the Gender Experiences Questionnaire (GEQ) and the Institutional Betrayal Questionnaire (IBQ) for novel uses, while maintaining strong reliability [3, 20]. We adapted the GEQ for use with all genders, and we adapted the IBQ to capture institutional betrayal at the climate level. Our study also boasts the first use of both questionnaires to assess high school experiences.

Two innovative findings stand out as the most important contributions of this study. First, identifying the relationship between high school gender harassment and college trauma-related symptoms provides support for the continued investigation of gender harassment as an impediment to healthy adolescent development. This finding suggests that gender harassment merits increased attention in high schools. Second, high school institutional betrayal’s association with college trauma-related symptoms above and beyond covariates plus gender harassment highlights the responsibility of schools to grapple with the potential harm caused by their indifference to harassment in their hallways. This finding extends the foundational research on institutional betrayal by (1) focusing on gender harassment and institutional betrayal in adolescence and (2) attempting to capture institutional betrayal at the climate level.

Two possible explanations may account for the finding that institutional betrayal did not moderate the relationship between gender harassment and trauma-related symptoms. First, the main effect of institutional betrayal may indicate that a high school’s indifference to gender harassment may hurt students regardless of whether the student experiences gender harassment directly. In other words, it is possible that a climate of tolerating gender harassment may harm students. Second, the IBQ only captures the negative end of a high school’s possible response to harassment, thus missing the potential benefit of a high school demonstrating institutional courage [31]. Expanding the scale to capture institutional courage might increase the likelihood of identifying a meaningful interaction.

### Limitations

Significant limitations constrain the conclusions drawn from this study. First and most importantly, we make no causal claims from these data because they are cross-sectional. Second, we did not account for possible confounders like childhood trauma and other types of high school sexual misconduct, which may reduce the share of trauma-related symptoms that are explained by gender harassment and institutional betrayal [32]. Third, though participants were blinded to the study’s contents and therefore unlikely to be biased by self-selection, we did draw from a sample of college students, which may introduce bias and severely limit generalizability [17, 33]. Fourth, our survey lacked attention checks, which may have inflated the reliability and degraded the validity of the data [34]. Finally, we used retrospective report to measure high school gender harassment and institutional betrayal. While evidence suggests that retrospective report of child abuse holds steady over time, retrospective report shares some of the drawbacks of cross-sectional designs, i.e., the inability to describe the natural history of a phenomenon [35].

### Implications

This study advances several important ideas with implications for educators. Gender harassment in high school may interfere with the important work of adolescence. Gender harassment and institutional betrayal may turn high school into a hostile work environment. Schools may attempt to address these issues by exercising institutional courage. Institutional courage includes strong, transparent policy that complies with criminal laws and civil codes, voluntary accountability and the willingness to apologize, sensitive responses to disclosure, self-study and regular anonymous surveys, and the commitment of resources to all of the above [36]. Students’ own suggestions parallel these recommendations [4].

### Future directions

In the near future, we plan to rerun this study with the addition of the AAUW sex-based harassment items, a measure of homophobic teasing, a measure of previous trauma exposure, and attention checks added in to replicate, extend, and strengthen these findings. More broadly, next steps should include longitudinal study designs with prospective recruitment to better describe the natural history of the phenomena. Additional next steps should include a social ecological approach to investigating the complex system in which gender harassment and institutional betrayal occur, including a focus on the student’s relationship to and expectations of the high school; this approach could provide important insight for designing interventions. Finally, the development and deployment of an institutional courage-based intervention for schools could support causal claims and healthy adolescent development.

## Supporting information

S1 Table Factor loadings on subscales of the Gender Experiences Questionnaire (Leskinen & Cortina, 2014; N = 535). For our factor analysis, we tested a 5-factor model, using maximum likelihood estimation with an oblique promax rotation, in order to be consistent with the original procedure used by Leskinen and Cortina (2014). In interpreting our model, we referred to benchmarks described in the literature to evaluate the fit. These benchmarks state that the fit is suitable when SRMSR is .10 or less, RMSEA is .08 or less, and TLI is .95 or higher (Williams et al., 2012). Results indicated that the SRMSR was excellent (.04), but the RMSEA value (.116) was higher than recommended at (90% CI[.109, .124]), and the TLI was lower than desired (.85). However, the low TLI value and high RMSEA value were consistent with the initial results found in Leskinen and Cortina (2014). In order to address this issue in the initial scale development, Leskinen and Cortina (2014) adjusted their fit in order to allow residuals for certain items to correlate with one another; this improved the model fit. Results of the factor analysis were remarkably consistent with result from Leskinen and Cortina (2014), with one exception. Item 17 (“Referred to the workplace as a ‘man’s space’ (e.g., women do not belong here)”) loaded onto work/family policing, instead of gender policing.
(PDF)

## References

[1] Dahl RE, Allen NB, Wilbrecht L, Suleiman AB. Importance of investing in adolescence from a developmental science perspective. Nature. 2018 2;554(7693):441–50. 10.1038/nature25770 29469094

[2] Leskinen EA, Cortina LM, Kabat DB. Gender harassment: Broadening our understanding of sex-based harassment at work. Law Hum Behav. 2011;35(1):25–39. 10.1007/s10979-010-9241-5 20661766

[3] Leskinen EA, Cortina LM. Dimensions of Disrespect: Mapping and measuring gender harassment in organizations. Psychol Women Q. 2014 3;38(1):107–23.

[4] AAUW. Crossing the line: Sexual harassment at school. Washington, DC: AAUW; 2011 52 p.

[5] Elliott DM, Briere J. Sexual abuse trauma among professional women: Validating the Trauma Symptom Checklist-40 (TSC-40). Child Abuse Negl. 1992 1 1;16(3):391–8. 10.1016/0145-2134(92)90048-v 1617473

[6] Gruber JE, Fineran S. The impact of bullying and sexual harassment on middle and high school girls. Violence Women. 2007 6;13(6):627–43. 10.1177/1077801207301557 17515409

[7] Stockdale MS, Logan TK, Weston R. Sexual harassment and posttraumatic stress disorder: Damages beyond prior abuse. Law Hum Behav. 2009;33(5):405–18. 10.1007/s10979-008-9162-8 19115099

[8] Sojo VE, Wood RE, Genat AE. Harmful workplace experiences and women’s occupational well-being: A meta-analysis. Psychol Women Q. 2016 3 1;40(1):10–40.

[9] Kingkade T. These teenagers say they were forced out of high school for reporting harassment [Internet]. BuzzFeed News. 2017 [cited 2019 Aug 12]. https://www.buzzfeednews.com/article/tylerkingkade/forced-out-of-high-school-for-reporting-harassment-lawsuits

[10] Kingkade T. Schools keep punishing girls—especially students of color—who report sexual assaults, and the Trump administration’s Title IX reforms won’t stop it [Internet]. 2019 [cited 2019 Aug 24]. https://www.the74million.org/article/schools-keep-punishing-girls-especially-students-of-color-who-report-sexual-assaults-and-the-trump-administrations-title-ix-reforms-wont-stop-it/

[11] Smith CP, Freyd JJ. Institutional betrayal. Am Psychol. 2014;69(6):575–87. 10.1037/a0037564 25197837

[12] Monteith LL, Bahraini NH, Matarazzo BB, Soberay KA, Smith CP. Perceptions of institutional betrayal predict suicidal self-directed violence among veterans Exposed to Military Sexual Trauma: MST and Perceptions of Institutional Betrayal. J Clin Psychol. 2016 7;72(7):743–55. 10.1002/jclp.22292 27007795

[13] Smidt AM, Rosenthal MN, Smith CP, Freyd JJ. Out and in harm’s way: Sexual minority students’ psychological and physical health after institutional betrayal and sexual assault. J Child Sex Abuse. 2019 3 11;1–15. 10.1080/10538712.2019.1581867 30856062

[14] Smith C. First, do no harm: Institutional betrayal and trust in health care organizations. J Multidiscip Healthc. 2017 4;10:133–44. 10.2147/JMDH.S125885 28435281 PMC5388348

[15] Dionisi AM, Barling J. It hurts me too: Examining the relationship between male gender harassment and observers’ well-being, attitudes, and behaviors. J Occup Health Psychol. 2018;23(3):303–19. 10.1037/ocp0000124 29927306

[16] Gillander Gådin K, Stein N. Do schools normalise sexual harassment? An analysis of a legal case regarding sexual harassment in a Swedish high school. Gend Educ. 2017 11;1–18.

[17] Rosenthal M, Freyd J. Sexual Violence on Campus: No evidence that studies are biased due to self-selection. Dign J Sex Exploit Violence. 2018 1;3(1). https://digitalcommons.uri.edu/dignity/vol3/iss1/7

[18] Cromer LD, Freyd JJ, Binder AK, DePrince AP, Becker-Blease K. What’s the risk in asking? Participant reaction to trauma history questions compared with reaction to other personal questions. Ethics Behav. 2006 10 1;16(4):347–62.

[19] Yeater E, Miller G, Rinehart J, Nason E. Trauma and sex surveys meet minimal risk standards: Implications for Institutional Review Boards. Psychol Sci. 2012 7 1;23(7):780–7. 10.1177/0956797611435131 22623507

[20] Smith CP, Freyd JJ. Dangerous safe havens: Institutional betrayal exacerbates sexual trauma. J Trauma Stress. 2013 2;26(1):119–24. 10.1002/jts.21778 23417879

[21] Smith CP, Freyd JJ. Insult, then injury: Interpersonal and institutional betrayal linked to health and dissociation. J Aggress Maltreatment Trauma. 2017 11 26;26(10):1117–31.

[22] R Core Team. R: A language and environment for statistical computing [Internet]. Vienna, Austria: R Foundation for Statistical Computing; 2018 https://www.R-project.org/

[23] Wickham H. tidyverse: Easily install and load the “tidyverse”. [Internet]. 2017. https://CRAN.R-project.org/package=tidyverse

[24] Revelle W. psych: Procedures for personality and psychological research [Internet]. Evanston, IL: Northwestern University; 2018. https://CRAN.R-project.org/package=psych

[25] Meyer D, Dimitriadou E, Hornik K, Weingessel A, Leisch F. e1071: Misc functions of the department of statistics, probability theory group [Internet]. 2019. https://CRAN.R-project.org/package=e1071

[26] Honaker J, King G, Blackwell M. Amelia II: A program for missing data [Internet]. 2011. http://www.jstatsoft.org/v45/i07/

[27] McWhite CD, Wilke CO. colorblindr: Simulate colorblindness in R figures [Internet]. 2019. https://github.com/clauswilke/colorblindr

[28] Wickham H. ggplot2: Elegant graphics for data analysis [Internet]. New York, NY: Springer-Verlag; 2016. http://ggplot2.org

[29] Willness CR, Steel P, Lee K. A meta-analysis of the antecedents and consequences of workplace sexual harassment. Pers Psychol. 2007;60(1):127–62.

[30] Committee on the Impacts of Sexual Harassment in Academia, Committee on Women in Science, Engineering, and Medicine, Policy and Global Affairs, National Academies of Sciences, Engineering, and Medicine. Sexual Harassment of Women: Climate, Culture, and Consequences in Academic Sciences, Engineering, and Medicine [Internet]. Johnson PA, Widnall SE, Benya FF, editors. Washington, D.C.: National Academies Press; 2018 [cited 2019 Aug 6]. https://www.nap.edu/catalog/24994 29894119

[31] Gómez JM, Smith CP, Gobin RL, Tang SS, Freyd JJ. Collusion, torture, and inequality: Understanding the actions of the American Psychological Association as institutional betrayal. J Trauma Dissociation. 2016 10 19;17(5):527–44. 10.1080/15299732.2016.1214436 27427782

[32] Walker HE, Freud JS, Ellis RA, Fraine SM, Wilson LC. The Prevalence of sexual revictimization: A meta-analytic review. Trauma Violence Abuse. 2019 1 1;20(1):67–80. 10.1177/1524838017692364 29333937

[33] Bornstein MH, Jager J, Putnick DL. Sampling in developmental science: Situations, shortcomings, solutions, and standards. Dev Rev DR. 2013 12;33(4):357–70. 10.1016/j.dr.2013.08.003 25580049 PMC4286359

[34] Abbey JD, Meloy MG. Attention by design: Using attention checks to detect inattentive respondents and improve data quality. J Oper Manag. 2017 11 1;53–56:63–70.

[35] Kendall-Tackett K, Becker-Blease K. The importance of retrospective findings in child maltreatment research. Child Abuse Negl. 2004 7 1;28(7):723–7. 10.1016/j.chiabu.2004.02.002 15261467

[36] Freyd JJ. When sexual assault victims speak out, their institutions often betray them [Internet]. The Conversation. 2018 [cited 2019 Aug 24]. http://theconversation.com/when-sexual-assault-victims-speak-out-their-institutions-often-betray-them-87050

