# Supplementary material for: Isn’t high school bad enough already? Rates of gender harassment and institutional betrayal in high school and their association with trauma-related symptoms
Source: PLoS One. 2020 Aug 19;15(8):e0237713. doi: 10.1371/journal.pone.0237713 (PMC7444512; doi:10.1371/journal.pone.0237713)
Supplement: S1 Table — For our factor analysis, we tested a 5-factor model, using maximum likelihood estimation with an oblique promax rotation, in order to be consistent with the original procedure used by Leskinen and Cortina (2014). In interpreting our model, we referred to benchmarks described in the literature to evaluate the fit. These benchmarks state that the fit is suitable when SRMSR is .10 or less, RMSEA is .08 or less, and TLI is .95 or higher (Williams et al., 2012). Results indicated that the SRMSR was excellent (.04), but the RMSEA value (.116) was higher than recommended at (90% CI[.109, .124]), and the TLI was lower than desired (.85). However, the low TLI value and high RMSEA value were consistent with the initial results found in Leskinen and Cortina (2014). In order to address this issue in the initial scale development, Leskinen and Cortina (2014) adjusted their fit in order to allow residuals for certain items to correlate with one another; this improved the model fit. Results of the factor analysis were remarkably consistent with result from Leskinen and Cortina (2014), with one exception. Item 17 (“Referred to the workplace as a ‘man’s space’ (e.g., women do not belong here)”) loaded onto work/family policing, instead of gender policing. (PDF) [file pone.0237713.s001.pdf]

S1 Table. Factor loadings on subscales of the Gender Experiences Questionnaire (Leskinen & Cortina, 2014; N = 535). For our factor analysis, we tested a 5-factor model, using maximum likelihood estimation with an oblique promax rotation, in order to be consistent with the original procedure used by Leskinen and Cortina (2014). In interpreting our model, we referred to benchmarks described in the literature to evaluate the fit. These benchmarks state that the fit is suitable when SRMSR is .10 or less, RMSEA is .08 or less, and TLI is .95 or higher (Williams et al., 2012). Results indicated that the SRMSR was excellent (.04), but the RMSEA value (.116) was higher than recommended at (90% CI[.109, .124]), and the TLI was lower than desired (.85). However, the low TLI value and high RMSEA value were consistent with the initial results found in Leskinen and Cortina (2014). In order to address this issue in the initial scale development, Leskinen and Cortina (2014) adjusted their fit in order to allow residuals for certain items to correlate with one another; this improved the model fit. Results of the factor analysis were remarkably consistent with result from Leskinen and Cortina (2014), with one exception. Item 17 ("Referred to the workplace as a 'man's space' (e.g., women do not belong here)") loaded onto work/family policing, instead of gender policing.

Items	Sexist Remarks	Crude/ Offensive behavior	Infantilization	Work/Family Policing	Gender Policing
1. Made sexist remarks about people of your gender	**0.75**	-0.04	0.07	0.07	0.03
2. Referred to people of your gender in insulting or offensive terms	**0.74**	-0.03	0.08	0.00	0.05
3. Made sexist remarks or jokes about women in your presence	**0.98**	0.00	-0.04	-0.04	0.02
4. Made sexist jokes in your presence	**0.97**	0.02	-0.06	-0.01	0.02
5. Said crude or gross sexual things in front of others or to you alone	0.36	**0.52**	-0.08	0.07	-0.05
6. E-mailed, texted, or instant messaged offensive sexual jokes to you	0.04	**0.80**	0.02	0.04	-0.07
7. Made unwanted attempts to draw you into discussion of sexual matters	-0.07	**0.91**	0.04	-0.03	-0.02
8. Told you stories of their sexual exploits when you did not want to hear them	0.00	**0.76**	0.01	-0.06	0.09
9. Displayed or distributed dirty pictures or stories (e.g., nude pictures)	-0.05	**0.80**	0.00	0.01	0.01
10. Talked to you as if you were a small child instead of speaking to you like an adult	0.03	0.01	**0.92**	-0.03	-0.01
11. Treated you as if you were stupid or incompetent	0.01	0.03	**0.91**	-0.02	-0.04

12. Publicly addressed you as if you were a child (e.g., dear, kid, etc.)	-0.02	0.00	**0.77**	0.07	0.06
13. Suggested women are better suited for raising children than being in the workplace	0.10	-0.04	0.02	**1.00**	-0.19
14. Suggested women belong at home, not in the workplace	0.05	-0.03	0.00	**1.04**	-0.18
15. Said employees who are mothers are less productive than other employees	-0.09	-0.01	-0.02	**0.68**	0.20
16. Said employees who are mothers are less dependable than other employees	-0.10	0.03	-0.01	**0.64**	0.25
17. Referred to the workplace as a ''man's space'' (e.g., women do not belong here)	0.04	0.06	0.01	**0.63**	0.08
18. Made you feel like you were less of a woman because you had traditionally masculine	-0.03	0.00	-0.04	0.04	**0.87**
19. Criticized you for not behaving ''like a woman should''	0.08	0.01	0.06	0.00	**0.75**
20. Treated you negatively because you were not ''feminine enough''	0.06	-0.01	-0.01	-0.08	**0.90**

Supplemental References:

Leskinen, E. A., & Cortina, L. M. (2014). Dimensions of disrespect: Mapping and measuring gender harassment in organizations. *Psychology of Women Quarterly*, 3*8*, 107-123. https://doi.org/10.1177/0361684313496549

Williams, L. J., Vandenberg, R. J., & Edwards, J. R. (2012). Structural equation modeling in management research: A guide for improved analysis. *The Academy of Management Annals*, *3*, 543–604.
