# Supplementary material for: Isn’t high school bad enough already? Rates of gender harassment and institutional betrayal in high school and their association with trauma-related symptoms
Source: PLoS One. 2020 Aug 19;15(8):e0237713. doi: 10.1371/journal.pone.0237713 (PMC7444512; doi:10.1371/journal.pone.0237713)
Supplement: Attachment — Submitted filename: response to reviewers.docx [file pone.0237713.s002.docx]

22 June 2020

Dear Dr. Castro-Alonso,

Thank you for taking the time to review our paper, “Isn’t high school bad enough already? Rates of gender harassment and institutional betrayal in high school and their association with trauma-related symptoms,” for publication in PLOS ONE. We appreciate the reviewers’ careful consideration of this article and their helpful suggestions and comments. We are excited to submit a revision of this manuscript, incorporating revisions suggested by the reviewers. We believe these revisions have strengthened the manuscript. Our responses to the reviewers’ feedback are below.

Sincerely,
Monika N. Lind, M.S.

Alexis A. Adams-Clark, M.S.

Jennifer J. Freyd, Ph.D.

| Reviewer Number | Reviewer’s Comment | Authors’ Response |
| --- | --- | --- |
| 1 | Title: I believe the authors should include the outcome of the study (i.e. traumatic symptoms) in the title, to summarize the focus of the article more explicitly. | This is an excellent suggestion, which we have implemented. |
| 1 | The authors need to provide more information regarding the hierarchical linear models (HLM) fitted. More specifically, the nested structure of the data that warrants the use of this modeling approach is not clearly explained (e.g. is it students nested within high schools, students nested within undergraduate courses). | The reviewer points out an important weakness in the reporting of our results. The term, “hierarchical,” lacks clarity, and many readers would likely have the same interpretation that the reviewer had. In this case, what we meant by “hierarchical regression” was a statistical test to show if variables of interest explain a statistically significant amount of variance in the dependent variable after accounting for all other variables. In other words, we did not use levels to nest our data. Unfortunately, because of the wide range of high schools that university students come from, it is outside the scope of the project to nest individual students within their respective schools in an MLM approach and investigate predictors at the school level. This is a limitation (and potential future direction) of this research. We have revised the text to clarify our approach. |
| 1 | While I understand that participants were not informed about the content of the study to avoid biased self-selection and that the study was approved by their institutional ethics’ committee, I believe it would be important to reflect on and discuss the inclusion of trigger warnings in future research and the risks of double victimization. | The reviewer rightly suggests that this research needs to be conducted with great care for participants. We have added a reference from our research group in the Procedure to provide evidence that trauma-related poses minimal risk to participants. |
| 1 | I believe that the sections Correlational results and Simple regression models are somewhat redundant as they both report on bivariate associations. The authors should focus more on reporting the HLM results. | The reviewer helpfully identifies an overlooked redundancy. We have condensed the correlational and simple regression results to include only the correlation table and the key bivariate association figures. |
| 1 | In pages 7-8, the authors state "Rather than reporting on experiences in the workplace, participants were instructed to report on experiences of gender harassing behaviors during high school by anyone associated with their high school, including classmates, coaches, teachers, school staff, and administrators." Should it also say "and themselves"? | We revised this sentence to improve its clarity. |
| 1 | In page 9, it says "All items were summed to create a total composite IBQ score ranging from 0 to 36, where higher scores represent more frequent institutional betrayal." If this was the case and there were missing data in some of the IBQ items, those cases would obtain lower scores. I believe averaging scores would be a better approach. | This is a reasonable concern, and we direct the reviewer to our Missing Data section on page 11, where we note the extremely small amount of missing data and our approach to imputation. When we scored the IBQ, data were already imputed so there were no items missing at that point. |
| 1 | As the authors suggest, the items of GEQ questionnaire differs somewhat by gender. I would suggest running reliability analysis separately to check that the internal consistency is similar among groups. | This is an excellent suggestion, which we have added to the paper. |
| 1 | Also, considering that some of the scales used were modified from their original versions and that they now refer to a different setting (high school instead of work place for GEQ), I would suggest running factor analysis and reporting on the factor loadings of the items in an appendix. | This is an excellent suggestion, which we have added to the paper. It’s especially valuable because it turns out that all but one item load very strongly onto the appropriate subscale factors -- a remarkable result for an adapted scale in a new population. |
| 2 | I would have liked to have seen a deeper consideration of the relationship between adolescents and high schools. [...] I would be interested to see how your line of research could start to shed light on the wide range of social structures and interpersonal interactions which I believe lie behind institutional betrayal. | This is a terrific insight, and we would love to see this research as well. We have added content to the Future Directions section to reflect these suggestions. |
